# Critical Challenges and Frontiers in Cartilage Tissue Engineering

**DOI:** 10.7759/cureus.53095

**Published:** 2024-01-28

**Authors:** Madhan Jeyaraman, Naveen Jeyaraman, Arulkumar Nallakumarasamy, Swaminathan Ramasubramanian, Sankalp Yadav

**Affiliations:** 1 Orthopaedics, ACS Medical College and Hospital, Dr MGR Educational and Research Institute, Chennai, IND; 2 Orthopaedics, Jawaharlal Institute of Postgraduate Medical Education and Research (JIPMER), Karaikal, IND; 3 Orthopaedics, Government Medical College, Omandurar Government Estate, Chennai, IND; 4 Medicine, Shri Madan Lal Khurana Chest Clinic, New Delhi, IND

**Keywords:** molecular therapies, nanotechnology, 3d printing, chondrocytes, cartilage tissue engineering

## Abstract

Cartilage tissue engineering has witnessed considerable advancements since its establishment in 1977, evolving from rudimentary surgical interventions to more nuanced biotechnological approaches. The field has navigated various challenges encompassing cellular considerations, scaffold material selection, environmental factors, and ethical and regulatory constraints. Innovations in cell source diversification, including chondrocytes, mesenchymal stem cells, and induced pluripotent stem cells, have been instrumental but not without their limitations, such as restricted cell proliferation and ethical dilemmas. Scaffold materials offer a unique dichotomy between natural substrates, which provide biocompatibility, and synthetic matrices, which grant mechanical integrity. However, translational hurdles in clinical applicability persist. Environmental factors, such as growth factors and thermal and mechanical forces, have been recognized as influential variables in cellular behavior and tissue maturation. Despite these strides, integration with host tissue remains a significant challenge, involving mechanical and immunological complexities. Looking forward, emerging technologies such as 3D and 4D printing, nanotechnology, and molecular therapies hold the promise of refining scaffold design and enhancing tissue regeneration. As the field continues to mature, a multidisciplinary approach encompassing thorough scientific investigation and collaboration is indispensable for overcoming existing challenges and realizing its full clinical potential.

## Introduction and background

Effective treatment approaches are urgently needed due to the rising prevalence of articular cartilage injuries brought on by circumstances such as trauma, biomechanical imbalances, and degenerative diseases [1]. Traditional approaches, such as microfracture and autologous chondrocyte implantation (ACI), have been shown to have serious drawbacks, including the inability to promote the body's healing mechanisms and the financial burden they place on healthcare systems [2]. Due to this, cartilage tissue engineering (CTE) has become well-known as a crucial field of regenerative medicine, using interdisciplinary skills in cell biology, biomechanics, and biomaterials research [3].

The importance of articular cartilage in joint health and mobility is widely understood since it allows for frictionless movement and force transfer between bones [4]. Although its avascular nature gives it a limited ability for regeneration, this complicates wound healing and promotes diseases such as osteoarthritis [1,2]. Modern research aims to create novel methods for the repair or replacement of injured cartilage to get beyond these inborn restrictions.

In the pursuit of these objectives, the field of CTE confronts several challenges. The area of CTE has several difficulties in achieving these goals. These span from cellular and material issues to environmental elements that impact tissue regeneration, as well as ethical and regulatory problems [5]. Despite these challenges, improvements in biomaterials present a viable way to tackle these problems [6]. This article aims to provide a thorough examination of the various aspects of CTE, with an emphasis on highlighting recent developments and promising future directions for overcoming the drawbacks of existing cartilage repair techniques.

## Review

Historical perspective

The area of CTE has seen tremendous change in its methodology, materials, and technical instruments since its start in 1977 [7]. The first methods for cartilage restoration included traditional surgical techniques, such as microfracture and ACI. These methods do, however, have significant drawbacks, such as donor site morbidity and variable long-term results [2]. Furthermore, non-surgical methods, including medication and physical therapy, have only temporarily reduced symptoms without regaining the functioning of native tissue [5].

Early studies in CTE focused on employing chondrocytes, the cartilage's resident cells, for repair treatments. This innovative technique was hampered by the restricted availability and poor proliferation rates of chondrocytes [7]. To overcome these issues, further research focused on alternative cell types such as mesenchymal stem cells (MSCs) and induced pluripotent stem cells (iPSCs). Because of their multipotency and increased proliferation rates, these cell types have shown great potential [2].

The continual evolution of the field has also been catalyzed by a deepening understanding of cartilage ultrastructure and biomechanics. Such advancements have propelled innovations in scaffold design, both natural and synthetic, to support cellular growth and function [5]. Recent years have witnessed a move toward scaffold-free systems and the incorporation of advanced technologies such as bioreactors, biosensors, and 3D bioprinting [7]. These technological advancements have been complemented by strides in biomaterials science and high-throughput analysis, thereby enabling the development of more sophisticated and effective strategies for cartilage repair [3].

**Figure 1:**
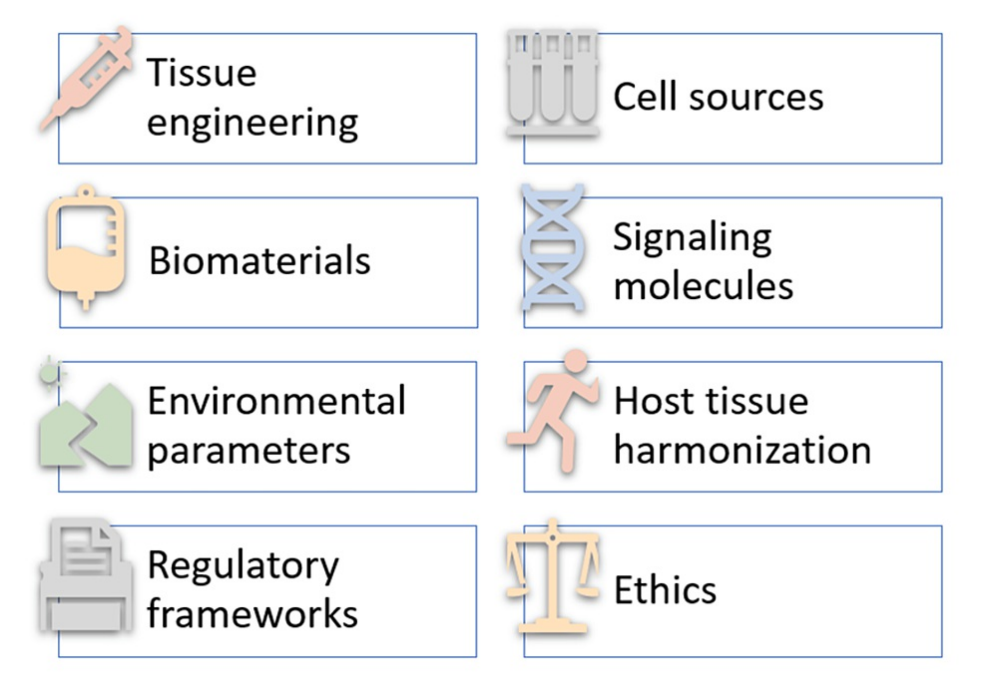
The strategies of CTE CTE: Cartilage tissue engineering Picture courtesy: Dr. Sankalp Yadav

CTE has evolved from its early reliance on traditional surgical techniques and chondrocyte-based interventions to a multi-faceted discipline enriched by advances in cell biology, biomechanics, biomaterials science, and technological innovations. These advancements, taken together, present intriguing pathways for overcoming the limits of previous techniques, ushering in a new era in the pursuit of successful and consistent cartilage regeneration. The strategies of CTE are depicted in Figure 1.

Cellular considerations

The selection of an appropriate cell type for CTE is critical to the success of regeneration techniques. Over the years, research has investigated a variety of cell sources, most notably chondrocytes, MSCs, and iPSCs, each with its own set of benefits and drawbacks.

Chondrocytes, the native cartilage cells, are an obvious candidate due to their capacity to create cartilaginous extracellular matrix. Their poor proliferative potential and proclivity for dedifferentiation during in vitro growth, on the other hand, pose substantial problems [8]. These constraints have prompted the investigation of alternate cell sources, such as MSCs and iPSCs. MSCs are especially intriguing because of their accessibility and multipotent differentiation potential, but they come with their own set of difficulties, such as ethical and regulatory concerns [1,2]. iPSCs, on the other hand, provide a theoretically limitless source of patient-specific cells that can be developed into chondrocytes while posing ethical and regulatory challenges [1].

The constraint imposed by cell senescence is one of the most important obstacles to cartilage tissue creation across cell types. This is particularly difficult for chondrocytes and MSCs, and it is aggravated by variables such as ex vivo expansion, donor age, and the presence of degenerative disorders [8]. To address this problem, many techniques have been implemented, including the use of growth factors, antioxidants, and serum deprivation. To prevent senescence, some studies advise keeping cells in a low-oxygen environment or using platelet-rich plasma [8].

Cell viability and proliferation are essential for the production of functional cartilage tissue and are determined by the cell type [2]. Furthermore, controlling the differentiation and maturation of these cells is critical for effective cartilage repair, particularly for stem cell choices such as MSCs and iPSCs [9]. Maintaining cellular phenotype while ensuring chondrogenic differentiation adds another degree of complication [9].

The selection of cell type for cartilage regeneration is a difficult yet crucial element, and researchers are confronted with various problems. These vary from particular limits connected with each cell type, such as mature chondrocytes' proliferative restrictions [1], to more general difficulties such as cell senescence and the necessity for regulated differentiation and maturation [8,9]. Therefore, a thorough knowledge of these issues is required for the advancement of efficient CTE technologies.

Biomaterial constraints

Material selection for scaffolds is an important factor in the successful creation of cartilage tissue, a process that requires biocompatibility, functionality, and mechanical properties [6]. For this goal, a variety of natural and synthetic materials have been investigated, each with its own set of characteristics [2].

Natural polymers such as collagen and chitosan are frequently used because of their inherent biocompatibility and biodegradability [9]. These materials readily promote cell proliferation and functioning, creating an environment conducive to tissue regeneration [5]. They may, however, lack the mechanical strength necessary for load-bearing applications, making them less suitable for some applications [2].

Synthetic materials, on the other hand, such as polycaprolactone (PCL) and poly(lactic-co-glycolic acid) (PLGA), provide better control over mechanical properties [2]. These materials may be created to imitate the elasticity and compressibility of native cartilage, which is an important factor to consider when developing functional synthetic tissues [10]. Despite their mechanical benefits, synthetic materials may pose biocompatibility and cell attachment problems, needing rigorous assessment before use [9].

Furthermore, new materials such as hydrogels and biostable polymers are emerging as intriguing alternatives that have the potential to revolutionize the area of tissue engineering [10]. These innovative materials attempt to fill gaps in both natural and synthetic choices, providing biological compatibility as well as mechanical tunability. Nonetheless, while these materials have shown promise in in vitro and animal model investigations, their translation to clinical settings remains a significant issue [1].

Scaffold material selection is critical in CTE since each material type has a unique profile of biocompatibility, biodegradability, and mechanical qualities. While natural materials are preferable in terms of biocompatibility, synthetic materials are superior in terms of mechanical tunability, and future advanced materials may bring the best of both worlds. However, the move from laboratory discoveries to clinical applications requires further research and development.

Microenvironmental parameters

In the realm of CTE, the local cellular environment within scaffolds and biomechanical cues serve as critical determinants for successful tissue regeneration. Among the essential components that influence this environment are growth factors, oxygen tension, nutrient supply, and mechanical forces.

Growth factors such as transforming growth factor beta (TGF-β), bone morphogenetic protein-2 (BMP-2), and insulin-like growth factor-1 (IGF-1) are instrumental in orchestrating chondrogenesis and cartilage repair [2,11]. These factors not only promote chondrocyte migration and proliferation but also enhance the chondrogenic differentiation of stem cells and recruit endogenous stem cells to the lesion area [11]. Despite their importance, challenges such as the risks of oncogenicity, abnormal differentiation, and effective delivery and retention within the scaffold persist [2]. Other key elements that contribute to cell viability and tissue formation include environmental circumstances, notably oxygen tension and nutrition supply [2]. Because cartilage is avascular, maintaining appropriate nutrition and oxygen diffusion is critical, as poor circumstances can negatively influence cell survival and differentiation.

Aside from biochemical signals, biomechanical considerations have a substantial impact on tissue properties and the quality of manufactured cartilage. Mechanical forces, such as compression and shear stress, are critical for guiding chondrogenic differentiation and functional tissue development [7,9]. Understanding mechanical qualities such as elasticity and poroelasticity is critical for customizing tissue engineering strategies [4].

Modifications to mechanical stimulation and the application of specific growth factor mixtures can also be used to direct cells toward chondrogenic lineages [7,9]. Thus, a comprehensive approach encompassing biochemical, mechanical, and environmental inputs is critical for effective cartilage tissue creation. For CTE to progress, an integrated knowledge of the roles and problems associated with growth factors, mechanical stresses, and environmental parameters such as oxygen tension and nutrition availability is required.

Host tissue harmonization

A key difficulty in the field of CTE is achieving seamless integration of synthetic cartilage constructions with natural host tissue. Despite tremendous progress in scaffold design and fabrication processes, the mechanical and immunological challenges of integration remain unresolved [11].

Ensuring the mechanical strength of the engineered-to-native tissue interface is one of the primary considerations in this endeavor. Poor integration can jeopardize both the newly treated tissue and the nearby original cartilage, emphasizing the importance of conducting comprehensive mechanical assessments to determine the structural integrity of the tissue interface [12]. Contemporary techniques have investigated several strategies for enhanced mechanical integration, such as the use of biomimetic scaffolds and surface changes. Furthermore, novel approaches such as 4D printing enable the creation of dynamically adjustable scaffolds, potentially improving mechanical compatibility [11].

Immunological compatibility between the modified cartilage and the host tissue is also critical. Immune reactions to implanted constructions can cause tissue rejection or deterioration of the engineered construct, causing problems for the repair's long-term stability and functionality [2]. Emerging techniques are investigating the modulation of cellular, material, and biomolecular components inside constructed scaffolds to alleviate these immunological difficulties. For instance, the inclusion of extracellular vesicles may offer a promising route to modulate the host immune response favorably [11].

While considerable strides have been made in CTE, the seamless integration of engineered constructs with native tissue remains a formidable challenge. To address this, future work must focus on enhancing mechanical compatibility and mitigating immunological challenges. This may involve a multidimensional approach that incorporates mechanical design, material science, and immunomodulatory tactics, potentially guided by advancements such as 4D printing and biomolecular manipulation [2,11,12].

Mechanical regulation and thermal challenges

In the multifaceted realm of CTE, addressing the intrinsic challenges posed by cartilage's avascularity, limited metabolic rate, and unique mechanical requirements stands as a focal point of research and development [13,14]. A paramount component that adds a layer of complexity to the engineering of functional cartilage tissue is the influence of mechanical stimuli, with tension emerging as a particularly significant factor. The influence of tension extends across cellular differentiation processes, molecular adaptations, and the functionality of engineered cartilage tissue [15]. Research indicates that tension, along with other mechanical forces such as compression and shear, significantly affects the formation of neo-cartilage from stem cells. The orchestrated application of these mechanical stimuli has been demonstrated to improve the structural and functional integrity of the engineered cartilage, rendering it more analogous to its natural counterpart [15].

In parallel with biological considerations, mechanical stimuli, including tension, have been integrated into bioengineering frameworks, most notably in the design of specialized bioreactors. These bioreactors aim to emulate physiological mechanical environments by applying carefully controlled mechanical forces to both cellular and tissue constructs. The resultant mechanical environment is deemed conducive to cartilage synthesis and maturation, providing a platform for the in vitro study and development of engineered cartilage [16].

On another front, the advent of nanotechnology has opened new avenues for incorporating thermal aspects into tissue engineering and regenerative medicine (TERM) [17]. Non-invasive thermal therapeutic modalities, such as photothermal therapy, magnetic thermotherapy, and ultrasound thermotherapy, are being actively explored. These therapies offer the potential for controlled thermal interventions, enabling more precise tissue regeneration parameters. While the initial findings are promising, ongoing research aims to define the parameters and conditions that are critical for the effective application of these thermal therapies, especially in the context of clinical translation [17].

Material science also makes a significant contribution to the evolving landscape of CTE. Advanced hydrogels, for instance, have been formulated to offer improved network crosslinking and the capacity for self-recovery post-damage in vivo [13]. These hydrogels serve a dual purpose: they act as structural scaffolds while also functionally mimicking the cellular microenvironment. Such a biomimetic approach facilitates the optimal conditions for new tissue growth and provides the mechanical attributes required for addressing specific mechanical forces, notably tension, that are inherent in both natural and engineered cartilage [13].

Current treatment strategies for articular cartilage defects range from microfracture techniques to autologous chondrocyte implantation. These methods, while displaying varying degrees of success, underline the challenges that persist in both the technical and regulatory domains of CTE. A comprehensive approach to resolving these challenges would inevitably necessitate both rigorous preclinical trials and detailed clinical evaluations to assess the effectiveness of these therapeutic strategies [18].

Thus, the intricate fabric of CTE is woven from multiple disciplinary threads, including biology, materials science, and engineering technology. Each of these disciplines offers unique insights and solutions but also presents its own set of challenges. The effective manipulation of mechanical stimuli, especially tension, in harmony with optimized thermal therapies and advances in biomaterial science constitutes the current focal point of research in the field. A comprehensive overview of key technical factors and challenges in CTE is tabulated in Table 1.

**Table 1:** A comprehensive overview of key technical factors and challenges in cartilage tissue engineering

Core Aspects	Subcategories	In-Depth Description of Technical Constraints and Opportunities
Cellular Considerations	Chondrocytes	Limited ability to proliferate and the tendency to dedifferentiate during in vitro growth, thus posing significant challenges for tissue engineering.
	Mesenchymal Stem Cells	Characterized by accessibility and multipotent differentiation potential, but encumbered by ethical and regulatory considerations.
	Induced Pluripotent Stem Cells	Offer a theoretically limitless source of patient-specific cells, but face ethical and regulatory hurdles.
	General Issues	Challenges include cell senescence, exacerbated by factors like ex vivo expansion and donor age, as well as the need for controlled differentiation and maturation.
Biomaterial Constraints	Natural Polymers	Highly biocompatible and biodegradable, but may lack mechanical strength for load-bearing applications.
	Synthetic Materials	Provides tunable mechanical properties but can pose challenges in biocompatibility and cell attachment.
	Advanced Materials	Emerging materials like hydrogels and biostable polymers aim to offer both biological compatibility and mechanical tunability.
Microenvironmental Parameters	Growth Factors	Essential for chondrogenesis and cartilage repair but comes with challenges like the risk of oncogenicity and abnormal differentiation.
	Environmental Conditions	The avascular nature of cartilage makes oxygen tension and nutrient supply critical for cell survival and differentiation.
	Mechanical Forces	Mechanical factors like compression and shear stress are crucial for guiding chondrogenic differentiation and functional tissue development.
Host Tissue Harmonization	Mechanical Challenges	Ensuring mechanical strength at the engineered-to-native tissue interface is vital for successful tissue integration.
	Immunological Challenges	Requires strategies to mitigate tissue rejection and enhance the long-term stability of the implanted engineered construct.
Mechanical and Thermal Challenges	Mechanical Stimuli	Mechanical forces, notably tension, significantly impact cellular differentiation, molecular adaptations, and the functionality of engineered tissue.
	Thermal Therapies	Exploration of non-invasive thermal interventions like photothermal therapy for controlled tissue regeneration.

Ethical and regulatory hurdles

The utilization of human and animal cells in CTE presents a landscape rich in ethical and regulatory complexities. Central to the ethical debate is the use of human embryonic stem cells (hESCs), derived from the inner cell mass of blastocyst stage embryos. These cells are unique in their pluripotent nature, capable of differentiating into various cell types, but their use raises significant moral and ethical concerns, particularly regarding the status of the embryo and the implications of embryo destruction [2,9]. The UK has been a pioneer in creating legislative frameworks for regulating hESC research, reflecting the global trend toward addressing these ethical issues through policy and law [19].

With the advent of biotechnological advancements, such as iPSCs, the ethical landscape of CTE has become even more multifaceted. iPSCs, created by reprogramming adult cells to a pluripotent state, offer an alternative to hESCs that potentially circumvents some of the ethical issues associated with embryo use. However, they introduce new challenges regarding the safety and long-term effects of such cells in clinical applications. Ensuring the ethical procurement of source cells for iPSCs and addressing issues related to genetic manipulation are critical concerns in this area [20].

Regulatory challenges in CTE are not confined to the laboratory but extend to the clinical application of cell-based therapies. Before these therapies can reach patients, they must undergo stringent preclinical and clinical testing to demonstrate safety and efficacy. This process is governed by complex regulatory frameworks that vary across different countries and regions, presenting challenges in terms of standardization and global collaboration. Moreover, scaling up production methods for clinical applications must comply with good manufacturing practices (GMP), further complicating the regulatory landscape [2]. Efforts to harmonize these regulations are crucial for facilitating the translation of research into clinical practice [3,5].

In addition to these scientific and technical challenges, ethical considerations extend to the principles of informed consent and privacy. The procurement of cells, whether from embryos, adults, or alternative sources such as umbilical cord blood or adipose tissue, requires a robust consent process that respects the autonomy and rights of donors. The privacy of donors, particularly in the case of iPSCs where genetic information is involved, must be safeguarded. Policies and guidelines are needed to address these concerns adequately, ensuring that the rights and welfare of all stakeholders are respected [1,9]. Furthermore, the field of CTE must grapple with issues of justice and accessibility. As these technologies advance, it is imperative to consider how they can be made available equitably, avoiding disparities in access to cutting-edge medical treatments. This consideration is particularly important in the context of global healthcare, where resource disparities can exacerbate inequities.

The ethical and regulatory challenges in CTE are dynamic and multifaceted. They encompass not only the sourcing and use of cells, particularly hESCs but also the broader implications of rapidly evolving biotechnologies and the intricacies of diverse regulatory frameworks. Addressing these challenges requires a comprehensive approach, involving ethical scrutiny, regulatory vigilance, and a commitment to equity and justice. As the field continues to advance, ongoing adaptation and consideration of both existing frameworks and emerging ethical implications are essential to ensure the responsible and beneficial progression of CTE [2,5].

Case studies

CTE has been the focus of several seminal studies that have not only illuminated its challenges but also offered innovative solutions (Table 2).

**Table 2:** Case studies in CTE (methodologies and insights) CTE: Cartilage tissue engineering

Author(s)	Focus Area	Key Methodologies	Significant Findings	Critical Analysis
Bakhshayesh et al. [2]	Therapeutic Avenues	Comprehensive review of the role of scaffolds, cells, and growth factors	Outlined the current state of the art and highlighted the importance of these elements in articular cartilage repair.	Valuable synthesis of existing knowledge; reiterates known concepts without introducing new experimental data
Lammi et al. [5]	Functional Cartilage Tissue	Various methodologies for cartilage tissue production, including scaffold-based and scaffold-free models	Elaborated on diverse methodologies and their respective advantages and disadvantages.	Provides comparative analysis but lacks experimental validation or clinical trials
Fang [1]	Cell Sources & Novel Biomaterials	Identification of suitable cell sources and development of novel biomaterials	Highlighted the pros and cons of different cell types and materials for effective cartilage repair.	Contributes understanding in material and cell source selection, but limited by its narrow focus
Stampoultzis [10]	Advancements in Mechanobiology & Materials	Detailed overview of biomaterial science, mechanobiology, and manufacturing processes	Emphasized the role of chondrocyte mechanobiology in the development of clinical-grade cartilage constructs.	In-depth understanding of mechanobiology; lacks practical application data
Jelodari et al. [11]	Scaffold-Cartilage Integration	Investigated 4D-printing, deployment of growth factors, and use of extracellular vesicles	Explored promising techniques for enhancing tissue integration with existing cartilage.	Innovative approach with 4D-printing; real-world application and scalability yet to be determined
Wei et al. [21]	Osteochondral Defect Regeneration	Use of stratified sodium alginate constructs combined with bioactive glass and other materials	Demonstrated successful outcomes in osteochondral defect regeneration.	Promising scaffold design integrating bioactive materials; long-term clinical efficacy needs further investigation
Zelinka et al. [7]	Clinical Efficacy	Implanting an esterified hyaluronic acid scaffold seeded with passaged chondrocytes	Resulted in the formation of hyaline-like cartilage in osteoarthritis patients.	Clinically relevant approach; larger-scale clinical trials needed for efficacy and safety confirmation

Bakhshayesh et al. presented a thorough review, capturing the current state of the art in articular cartilage repair. Their focus on scaffolds, cells, and growth factors in therapeutic avenues highlighted the interplay between these components as crucial for effective repair strategies [2]. This comprehensive synthesis, while reiterating established concepts, did not venture into new experimental territories.

Further extending this knowledge base, Lammi et al. discussed various methodologies for producing functional cartilage tissue. Their analysis included both scaffold-based and scaffold-free models, providing a comparative overview but lacking direct experimental validation or clinical trial data [5]. This study, therefore, stands as a theoretical framework rather than a practical guideline for cartilage tissue engineering.

Fang contributed significantly by examining the challenge of identifying suitable cell sources and developing novel biomaterials for cartilage repair. This study highlighted the advantages and limitations of different cell types and materials, offering a detailed perspective on material and cell source selection, albeit with a limited broader context [1]. Stampoultzis et al. added another dimension to this narrative by delving into the advancements in biomaterial science, mechanobiology, and manufacturing processes. The emphasis on chondrocyte mechanobiology for developing clinical-grade cartilage constructs provided an in-depth understanding of this aspect, though it lacked data on practical applications [10].

Jelodari et al. introduced a novel layer of complexity by focusing on scaffold-cartilage integration. Their exploration of 4D printing, growth factor deployment, and extracellular vesicles opened new avenues for enhancing tissue integration. This innovative approach signaled a shift toward more advanced technologies, although the practicality and scalability of these methods in real-world applications remain to be seen [11].

In the realm of osteochondral defect regeneration, Wei et al. demonstrated a significant leap forward. They successfully employed stratified sodium alginate constructs, combined with bioactive glass and other materials, to achieve promising regeneration outcomes. This approach, integrating bioactive materials in scaffold design, signifies a substantial advancement towards mimicking natural tissue complexity, although long-term clinical efficacy requires further exploration [21].

Zelinka et al. provided a critical applied perspective through their study on the clinical efficacy of implanting esterified hyaluronic acid scaffolds seeded with passaged chondrocytes. This method led to the formation of hyaline-like cartilage in osteoarthritis patients, presenting a viable strategy for cartilage repair. However, large-scale clinical trials are necessary to fully establish its efficacy and safety [7].

Collectively, these research endeavors have significantly expanded our understanding of CTE. They have addressed challenges ranging from scaffold design and cell source selection to mechanobiology and material integration. The methodologies and insights derived from these studies serve as a valuable framework for future research in the field.

Future prospects

Due to rapid advancements in a variety of disciplines, the future of CTE is becoming increasingly optimistic, necessitating collaborative efforts from clinicians, researchers, and industrial partners for therapeutic progress [2]. Emerging technologies such as 3D and 4D printing, nanotechnology, bioreactors, and biosensors are key to these breakthroughs. These technologies provide unparalleled accuracy in scaffold design, as well as the possibility of dynamic interaction with their biological environment [11].

For instance, 4D printing technology enables 3D-printed constructions to adapt in real time to their surroundings, adding complexity and usefulness to previous approaches [11]. Concurrently, advances in material science are producing innovative biomaterials that dramatically improve cell growth and differentiation, such as esterified hyaluronic acid scaffolds seeded with passaged chondrocytes [7,10]. These biomaterials not only serve as more effective substrates, but they can also include bioactive compounds, encouraging tissue regeneration even further [2].

Moreover, there is a rush in research into sophisticated molecular treatments that target co-resident stem and progenitor cells, as well as the use of decellularized extracellular matrices for scaffold development [7,8]. These tailored techniques are expected to complement larger advancements in stem cell research, gene editing, and personalized medicine, all of which have the potential to greatly improve the efficacy of cartilage repair and regeneration efforts [2].

While these emerging technologies undeniably hold significant promise, continued research must focus on elucidating the underlying mechanisms governing these innovative therapeutic modalities [7]. A nuanced understanding of these mechanisms will not only validate the efficacy of these approaches but also accelerate the development of more refined and effective therapies for CTE.

The combination of diverse skills and cutting-edge technology is laying the groundwork for game-changing advances in CTE. However, further study is required to fully realize these technologies' and treatment methods' revolutionary potential [7].

## Conclusions

CTE presents a complex array of challenges that demand a multidisciplinary approach for meaningful clinical translation. Key factors include selecting the most suitable cell type, ensuring cell viability, and controlling proliferation and differentiation for successful tissue regeneration. Overcoming challenges in biomaterial choice and mechanical properties requires close collaboration between materials scientists and clinicians to achieve an optimal balance between material strength and biocompatibility. Environmental factors, including growth factors, oxygen levels, nutrient supply, and mechanical forces, significantly impact cell behavior and tissue engineering success. Integrating engineered tissue with host tissue remains challenging, necessitating innovative strategies for scaffold-tissue interfaces, immunological concerns, and long-term stability post-implantation. Ethical and regulatory considerations add further complexity, emphasizing the importance of strict adherence to guidelines for safety and efficacy in engineered cartilage products. Ongoing research and collaborative efforts among clinicians, researchers, and industry partners are essential to surmount these multifaceted challenges, propelling CTE closer to clinical applications, amidst the balance of promise and obstacles.
